# Water Quality of Urban Streams: The *Allium cepa* Seeds/Seedlings Test as a Tool for Surface Water Monitoring

**DOI:** 10.1155/2014/391367

**Published:** 2014-12-10

**Authors:** Camila Gonçalves Athanásio, Daniel Prá, Alexandre Rieger

**Affiliations:** ^1^Department of Biology and Pharmacy, University of Santa Cruz do Sul, Avenue Independência 2293, 96815-900 Santa Cruz do Sul, RS, Brazil; ^2^Postgraduation Course of Health Promotion, Department of Biology and Pharmacy, University of Santa Cruz do Sul, Avenue Independência 2293, 96815-900 Santa Cruz do Sul, RS, Brazil; ^3^Laboratory of Biotechnology and Genetics, Department of Biology and Pharmacy, University of Santa Cruz do Sul, Avenue Independência 2293, 96815-900 Santa Cruz do Sul, RS, Brazil

## Abstract

The present study investigates the genotoxic, mutagenic, and cytotoxic potential of surface waters in urban streams using *Allium cepa* and analyzes the applicability of this assay for environmental monitoring. Water samples were collected from three streams located in the urban area of a municipality in the south of Brazil. For each stream, two samples were collected, one upstream and one downstream of the pollution discharge site. Physicochemical evaluation indicated that all samples had various degrees of environmental impact, but substantial impact was seen for the downstream samples of the Preto and Pedras streams. All samples increased the frequency of chromosome aberrations (*P* < 0.05). The sample from Pedras downstream site also caused a decrease in mitotic index (*P* < 0.08) and increase in micronuclei (*P* < 0.08) frequency, indicating potential cytotoxicity and mutagenicity. The Pedras stream receives mixed industrial and urban wastewater, while the Lajeado and Preto streams receive wastewater predominantly domestic in nature, which may partially explain the difference in toxicity among the samples. Moreover, the *Allium cepa* seeds/seedlings were shown to be extremely sensitive in detecting the genotoxicity of environmental water samples and can be applied as the first tool for environmental health hazard identification and prediction.

## 1. Introduction

Streams located near urban areas are considered the major source of water to the population in the urbanized area. Domestic, industrial, and agricultural wastewaters are discharged into those rivers and streams, causing deterioration of the environment. This deterioration can cause great problems for human health as well as for aquatic flora and fauna [1, 2].

Domestic sewage discharge leads to more problems in water quality due to its typically large volume and high amounts of organic charge and other contaminants, such as oil, metals, and pesticides. Additionally, a diversity of organic compounds carried to water sources as the consequence of human activities can be persistent in the environment and be transported long distances, affecting regions in which they were never produced [3, 4].

The classical approach for water quality assessment is to combine physical, chemical, and microbiological methods to obtain complete results about the composition of the water sample. However, this merely characterizes the sample in a snapshot, neglecting the changes that naturally occur in a dynamic environment, such as streams and rivers, and the possible effects of exposure to these substances on aquatic organisms and human health.

Many toxic compounds have the ability to interact with genetic material, which can cause alterations or damage to DNA, and, thus, are termed genotoxic or mutagenic agents [5]. The presence of many contaminants in the environment has been shown to cause health problems, including some types of cancer [6]. Thus, it is extremely important that genotoxic and mutagenic assays are employed for environmental monitoring and risk assessment to identify the effects of pollutants on aquatic organisms and human health, complementing the physical, chemical, and microbiological analyses [7].

The* Allium cepa* test is a fast and sensitive assay to detect environmental genotoxins and mutagens. It allows the assessment of several endpoints, such as micronuclei formation, chromosome aberration, and mitotic index, and allows the evaluation of the mechanism of action of the pollutants on the DNA of exposed organisms. The* Allium* test was introduced by Levan [8, 9] and later applied for environmental monitoring by Fiskesjö [10]. The US-EPA Gene-Tox Program classified it as one of the most well established plant assay systems for environmental monitoring [11]. This test system has recently been used for the toxicity evaluation of chemical compounds, nanomaterials, phytochemicals, and environmental samples [12–17].

Additionally, this test is an important method for screening environmental pollution and is useful for assessing the water and sediment quality of rivers [18–21]. The use of* A. cepa* seeds provides an advantage over the use of bulbs due to the homogeneity of* A. cepa* seeds. Moreover, results of this test can help indicate the real health hazard of toxins because the fixation of errors during cell division and the toxicity caused by those damages can interfere in the development of organisms, affecting them as a whole and even affecting the local biota [22, 23].

The aim of this study was to investigate the genotoxic, mutagenic, and cytotoxic potential of surface waters in three urban streams with different sources of contamination using the* Allium cepa* seeds/seedlings test and to analyze the applicability of this assay to environmental monitoring and health hazard determination of surface waters.

## 2. Material and Methods

### 2.1. Collection Sites and Water Sampling

**Figure 1:**
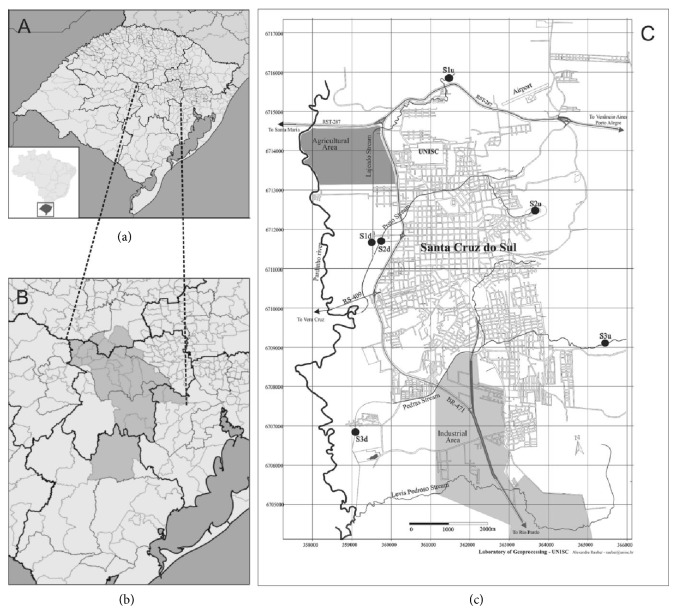
Location of the sampling sites within the urban area of Santa Cruz do Sul. (a) Rio Grande do Sul State; (b) Vale do Rio Pardo Region; and (c) urban area of Santa Cruz do Sul; (S1u and S1d) Lajeado stream; (S2u and S2d) Preto stream; and (S3u and S3d) Pedras stream.

In August 2011, during the winter (cold and wet season), water samples were collected at three streams (Lajeado, Preto, and Pedras streams) located in an urban area of Santa Cruz do Sul, RS, Brazil (Figure 1). Santa Cruz do Sul municipality is located in Vale do Rio Pardo in the central area of Rio Grande do Sul, the southernmost Brazilian State. Its Human Development Index (HDI) is 0.773 [24], and its economy is based on tobacco production, processing, and sale, as well as on trade and services and, to a minor extent, industry. The city has distinct areas of domestic and industrial occupation (as shown in the map) contributing to the differences in the composition of sewage discharged into various streams.

The Pardinho River is responsible for the city's water supply and receives contributions from many streams, including the Lajeado stream, Preto stream, and Pedras stream. These three streams are impacted by domestic and industrial sewage from the urban area of the city and from areas contaminated by agricultural practices, which degrade the water quality downstream of the city [25].

Each stream was sampled at two sites: one located upstream of the urbanized area of the municipality and another immediately downstream of the site affected by urban pollution. The collection sites S1u and S1d were in the Lajeado stream, S2u and S2d were in the Preto stream, and S3u and S3d were in the Pedras stream.

In the course of a single day, water temperature was taken in situ and water samples were collected from the sites, stored in thermal boxes, and transferred to the lab. In the lab, conductivity (*μ*S/cm), pH, and turbidity (NTU) were measured, together with dissolved oxygen (mg L^−1^). Dissolved oxygen was measured by the Winkler method according to APHA [26].

### 2.2. *Allium cepa* Seeds/Seedlings Test

Assays were carried out using seeds of* Allium cepa*, Baia Piriforme variety (98% expected germination), that were untreated with pesticides and imported from Italy by ISLA (Porto Alegre, RS, Brazil). Seeds were germinated in Petri dishes containing water from the collection sites. The seeds/seedlings were allowed to grow at 25°C for 5 days. Control tests were performed using ultrapure water as the negative control (NC) and 3 mg L^−1^ copper sulfate as the positive control (PC). Copper sulphate is a compound with known effects on* A. cepa* root cells, presenting both cytotoxicity and genotoxicity [27].

After exposure, the number of seeds germinated was counted, and 10 root tips were randomly removed and fixed in 3 : 1 methanol/glacial acetic acid for 24 h at 4°C. Then, the root tips were placed in 70% ethanol and stored at 4°C until analysis.

Microscope slides were prepared by squashing the root tips. In brief, root tips were washed in distilled water and hydrolyzed in 1 N HCl for 30 min. Then, the meristematic regions were cut and dismantled in 2 drops of 2% acetic orcein solution. Ten roots were analyzed for each collection site, along with NC and PC. Cytotoxicity (mitotic index (MI)) and clastogenicity/aneugenicity (micronuclei (MN)) were evaluated in 500 cells per slide, totaling 5000 cells per surface water/control sample. In each slide, chromosomal aberrations (CA), considered to be the presence of C-metaphase cells, chromosome bridges, chromosome losses, and chromosome breaks were quantified in 100 mitotic cells (cells in metaphase, anaphase, or telophase). The aberrations were classified as a single endpoint, following the criteria used by Leme and Marin-Morales [28]. The differences between the control and each collection site and between the upstream and downstream sites of the same stream were determined statistically by the Mann-Whitney *U* test (*P* < 0.05).

## 3. Results and Discussion

### 3.1. Physical and Chemical Characterization

The physical and chemical parameters obtained from water samples are presented in Table 1.

Electrical conductivity and turbidity were the parameters that showed values in disagreement with the guidelines of water quality for freshwater ecosystems. Electrical conductivity values were higher in the downstream than upstream collection sites of the same stream. S2d and S3d presented higher values than the other collection sites, 163 *μ*S/cm and 105 *μ*S/cm, respectively. This parameter is useful for the evaluation of water quality because it can indicate the quantity of salts in the water and represents an indirect measure of pollutant concentration. In addition, increased values of conductivity can happen due to the presence of chlorine, phosphates, and nitrates from sewage discharges [29]. The values obtained did not indicate the real state of water quality degradation, most likely due to dilution effects from rainfall; however, S2d and S3d showed values over 100 *μ*S/cm, which, according to CETESB [30], characterizes impacted environments.

With regard to turbidity, even though the samples presented values above the limit, the effects of the raining period cannot be discarded. The rainfall should also be considered when analyzing the dissolved oxygen values. All the collection sites presented high values for this parameter, although some of them were notably impacted by organic pollution, which is known to deplete oxygen levels in water. Data obtained from the Meteorological Center of the University of Santa Cruz do Sul indicated a historical average rainfall for August of 160 mm, and, in August 2011, the accumulated rainfall was 206 mm, nearly 30% higher than the average rainfall for this region in that month. Thus, the samples suffered dilution effects, and the results of the present work are valuable for periods with similar characteristics.

It should be noted that the streams in this study had never been analyzed with a genotoxic or mutagenic perspective. The available studies for the same collection sites are focused on an ecological and ecotoxicological perspective. Using diatoms as bioindicator organism, higher pollution levels were detected specially in the lower reaches; however the upper reaches were also contaminated. High values for phosphate, biochemical oxygen demand after 5 days, and thermotolerant coliforms were found, and the phosphate levels were responsible for the classification of these streams at the poorest water quality level [31, 32]. Another study, evaluating acute and sublethal effects of water and sediment samples on* Ceriodaphnia dubia,* showed strong water quality degradation caused by industrial and domestic discharges [33].

### 3.2. Cytotoxic and Genotoxic Potential of the Water Samples

The germination level did not differ between seeds exposed to surface water samples/controls. Values varied from 73% to 91% of seeds germinated (individual data not presented).

**Figure 2:**
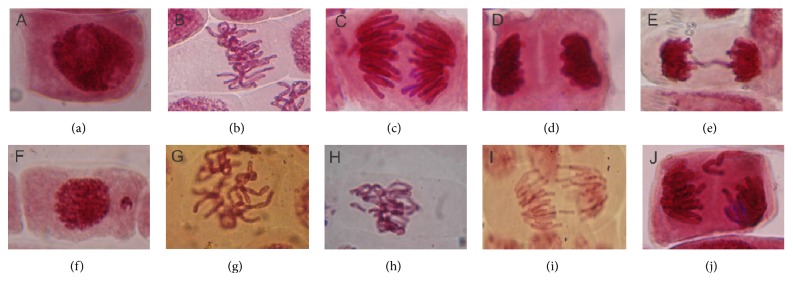
*Allium cepa* meristematic cell. (a) Normal interphase cell. (b) Normal metaphase cell. (c) Normal anaphase cell. (d) Normal telophase cell. (e) Telophase cell with bridge. (f) Interphase cell carrying micronuclei. (g) C-metaphase cell. (h) Metaphase cell with chromosome break. (i) Anaphase cell with chromosome break. (j) Anaphase cell with chromosome loss.

Table 2 presents the values of the mitotic indices, micronuclei, and chromosomal aberrations for the collection sites and controls. Some of the chromosomal aberrations found in* A. cepa* cells after exposure to the samples are exemplified in Figure 2.

Microscopic analysis of* A. cepa* root tips exposed to the different water samples revealed an impact on the cell cycle, with induction of CA, MN, and MI alterations from the period that the water samples were obtained. The MI and MN values of seeds exposed to the samples did not differ significantly from the NC, but those exposed to samples from the Pedras downstream site had a nearly significant decrease in MI (*P* < 0.08) and increase in MN (*P* < 0.08), indicating potential toxicity.

According to Leme and Marin-Morales [18], the MI, characterized by the number of dividing cells, has been used as a parameter to assess the cytotoxicity of several agents. The decrease in MI values can indicate impacts of chemicals on the growth and development of exposed organisms. Also, increase in the cell division, which can be harmful to the cells, can be used to estimate cytotoxic effects of contaminants. Both increase and reduction of MIs can be useful indicators of pollution in environmental monitoring (reviewed by Leme and Marin-Morales [18]). The MI values of the samples were close to that found for the NC, with the exception of the PC, which had a statistically significant lower MI than the NC (*P* < 0.01). Although it was not statistically significantly different from NC, the S3d sample also showed some decrease in the number of mitotic cells, indicating cytotoxic effects of this sample. Moreover, when comparing with the upstream site (S3u) it presented a lower MI value (*P* < 0.01).

MN is a parameter used to evaluate mutagenic potential of samples because it is the result of the absence or incorrect repair of alterations in the parent cells. It is considered by many authors as the most effective and simplest endpoint to analyze. According to Leme et al. [34], large MN indicates an aneugenic effect resulting from a chromosome loss, whereas small MN may indicate a clastogenic action resulting from a chromosome break. To corroborate the sensitivity of MN in our test system, the MN frequency of the PC was 7-fold higher than that of the NC (*P* < 0.05). MN did not differ between the collection sites and the NC, despite the fact that the S3d collection site had an increased MI.

For both parameters, MI and MN, the collection sites of the Pedras stream showed a significant difference compared between themselves; MI was higher in S3u than in S3d (*P* < 0.01). For MN, S3d showed the highest values of all collection sites and differed statistically from S3u (*P* < 0.05).

When comparing sites S2d and S3d, even though electrical conductivity was increased at both sites, indicating a potential sewage contamination, the MN frequency of S2d was lower than that of S3d. This result suggests that the water composition differs between the streams due to the different sources of pollution, as shown in Figure 1. Despite the need for additional studies of collection sites to identify the sources of contamination with cytotoxic and mutagenic potential, the* A. cepa* test is a useful tool for establishing priority sites for further analyses.

Chromosomal aberrations are a useful endpoint for the detection of the genotoxic effects of toxic agents and also enable the assessment of clastogenic and aneugenic actions.

In a simple way, CA, such as chromosome bridges and breaks, are indicators of a clastogenic action, whereas chromosome losses, delays, adherence, multipolarity, and C-metaphases result from aneugenic effects [18, 35]. For this study, it is clear that the total CA parameter was the most sensitive parameter for the evaluation of water quality at the collection sites. The PC differed statistically from the NC (*P* < 0.001), validating the test. The S1u and S1d samples also presented differences (*P* < 0.01), indicating alterations along the stream course in the urban area. It is important to note that the alterations identified as CA could lead to the formation of MN in the daughter cells when the damage is not repaired [18].

Evaluating the total CA, all collection sites showed increased values compared to the NC, and the downstream sites also presented higher values than the upstream sites. Thus, it is possible that all collection sites have high levels of genotoxicity.

For the three streams studied, the anthropogenic effects caused by sewage discharges are clear. Due to their locations, displayed in Figure 1, the streams receive different wastewater composition depending on the activities occurring along their watersheds. The Lajeado stream is impacted by domestic sewage and can receive waste from agricultural areas located close to the upstream collection site (S1u) and in the course of stream. The Preto stream is part of a microbasin that receives surface runoff and domestic sewage from a large area of the city. Different from the other streams, the Pedras stream is located near an industrial area, most likely receiving industrial sewage rather than domestic effluent.

Thus, based on the results presented in Table 2, it can be inferred that domestic sewages show lower mutagenic potential because the Preto stream samples did not increase the MN values in the* A. cepa* cells, while the Pedras stream samples caused a nearly statistically significant increase in the MN value in the* A. cepa* cells. These results indicate that industrial sewage can carry toxic compounds to the Pedras stream that has mutagenic, genotoxic, and cytotoxic potential. The presence of agricultural areas near the course of the Lajeado stream could be responsible for the observed increase in CA, but future studies on these collection sites are necessary to determine the real contribution of different effluents in water quality degradation.

Despite the fact that genotoxic and mutagenic assays do not identify the pollutants present in water samples, they are able to diagnose the possible effects of those pollutants, which is not possible using the classical environmental monitoring approach. The* A. cepa* test is cheap and fast, enables the identification of health hazards in water samples, and is suitable for use in environmental monitoring. According to Liman et al. [36], the* A. cepa* test is more sensitive than the Ames test, the traditional assay for mutagenic potential identification.

The* A. cepa* test is intended to be an inexpensive environmental monitoring tool for the initial screening of water samples. Once the* A. cepa* test identifies a site as a potential toxic risk for organisms, the sample from that site can be analyzed more completely using chemical analyses, which are usually expensive, to characterize and quantify the pollutants present in the water sample. Tabrez et al. [37] also suggested the use of the* A. cepa* test as the first bioassay for estimation of surface water mutagenicity.

It is important to emphasize that these three urban streams had not been previously analyzed for genotoxic, mutagenic, or cytotoxic potentials. The streams are important tributaries of the Pardinho River, the primary source of water for Santa Cruz do Sul. As shown in a previous study performed in 2009 by our group, native fish of Rio Pardinho had increased frequencies of MN when living in areas of the river impacted by urban pollution [38]. This result indicates that the environmental samples of the river possessed genotoxic compounds, as evaluated in different biomonitors. The results of the present study indicate that the genotoxic potential of the Rio Pardinho River may be due to its stream tributaries, although each one has a different genotoxic load. Determining the relative contribution of each tributary might aid public authorities in the identification of specific pollution sources and the development of strategies to mitigate genotoxic risk.

## 4. Conclusion

Based on the findings presented, the deterioration process of water quality caused by sewage discharges in these urban streams is clear.

The* Allium cepa* seeds test was useful as a hazard recognition tool for surface water samples and could be successfully used as a primary approach for environmental monitoring.

With regard to the analyzed streams, the increase in CA evaluated using* Allium cepa* seeds demonstrated, at least, the genotoxicity of the water samples and their potential to cause DNA damage. The significant effects on MN and MI indicate potential alterations to the cell cycle, compromising water quality and health. Nontreated domestic and industrial sewage are the major sources of pollution in urban areas and should not be tolerated because Santa Cruz do Sul is a developed municipality with a high HDI and a healthy economy.

It should be noted that the raining period may have influenced the results by diluting the samples, decreasing their genotoxic, mutagenic, and cytotoxic potential. However, the* Allium cepa* test still had enough sensitivity to indicate the toxic hazard of the samples and to guide the next level of monitoring using particular chemical analyses to identify the compounds in the water samples responsible for the toxic potential presented.

## Figures and Tables

**Table 1:** Physical and chemical values from different collection sites of urban streams in Santa Cruz do Sul.

	Lajeado stream		Preto stream		Pedras stream		CONAMA
	S1u	S1d	S2u	S2d	S3u	S3d	357/2005—class 1
Temperature (°C)	12	12	13	14	12	13	—
pH	6.85	6.67	6.60	6.82	6.76	6.72	6.00–9.00
EC (*µ*S/cm)	66.5	86.7	51.0	163.3	78.8	105.5	100^1^
DO (mg L^−1^)	9.8	9.2	9.6	8.2	9.6	8.2	6
Turbidity (NTU)	67.8	66.7	38.2	32.8	59.1	52.9	40

^1^Values established according to CETESB (2005). EC: electric conductivity; DO: dissolved oxygen.

**Table 2:** Mean and standard deviation of chromosomal aberrations (CA), micronuclei (MN), and mitotic index (MI) observed in meristematic cells of *A. cepa* exposed to water samples of Lajeado, Preto, and Pedras Streams.

Samples		MI	MN	CA
Controls	NC	16.36 ± 3.95%	0.4 ± 1.26	13.0 ± 6.75
	PC	11.44 ± 2.76%^*^	2.8 ± 3.01^*^	45.0 ± 15.81^*^
Lajeado stream	S1u	14.54 ± 3.56%	0.2 ± 0.63	32.0 ± 12.29^∗c^
	S1d	14.48 ± 1.80%	1.0 ± 1.41	58.0 ± 13.17^∗c^
Preto stream	S2u	14.42 ± 1.27%	0.2 ± 0.63	26.0 ± 17.76^*^
	S2d	14.74 ± 3.39%	0.6 ± 0.97	37.0 ± 18.89^*^
Pedras stream	S3u	17.36 ± 2.64%	0.2 ± 0.63	33.0 ± 21.11^*^
	S3d	13.32 ± 3.10%^a^	2.0 ± 2.67^b^	48.0 ± 33.27^*^

MI: mitotic index expressed in % of dividing cells; MN: micronucleus in 1000 cells; and CA: chromosomal aberration in 1000 dividing cells. PC: 3 mg L^−1^ CuSO_4_.  ^*^Statistically significant results (*P* < 0.05) compared to NC; ^a^statistically significant difference between S3u and S3d comparing MI values (*P* < 0.01); ^b^statistically significant difference between S3u and S3d comparing MN values (*P* < 0.05); and ^c^significant difference between S1u and S1d comparing CA values (*P* < 0.01).
